# In Rats, Whole and Refined Grains Decrease Bone Mineral Density and Content through Modulating Osteoprotegerin and Receptor Activator of Nuclear Factor Kappa B

**DOI:** 10.3390/biomedicines11061686

**Published:** 2023-06-10

**Authors:** Hussein Sakr, Zenat Khired, Marzieh Moghadas

**Affiliations:** 1 Department of Physiology, College of Medicine and Health Sciences, Sultan Qaboos University, Muscat 123, Oman; marziemoqadass@gmail.com; 2 Surgical Department, Jazan University, Jazan 45142, Saudi Arabia; zkherd@jazanu.edu.sa

**Keywords:** osteoporosis, grain, bone mineral density, RANK

## Abstract

Wheat is a staple grain in most parts of the world and is also frequently used in livestock feed. The current study looked at the impact of a wheat grain diet on bone turnover markers. Thirty male rats (*n* = 10) were separated into three groups of ten. The rats in Group 1 were fed a chow diet, while the rats in Group 2 were provided whole grains. The rats in Group 3 were fed refined grains. Each rat’s bone mineral content (BMC) and bone mineral density (BMD) were measured after 12 weeks in the tibia of the right hind limb. We also looked at the amounts of bone turnover indicators in the blood. TRAP-5b (Tartrate-resistant acid Phosphatase 5b), NTx (N-telopeptide of type I collagen), DPD (deoxypyridinoline), alkaline phosphatase (ALP), and osteocalcin (OC), as well as the levels of Receptor Activator of Nuclear Factor Kappa B (RANK) and osteoprotegerin (OPG). Rats fed whole and refined grains showed lower BMC and BMD (*p* < 0.05) than the control group rats. The grain diet resulted in lower OPG, OC, and ALP levels than the chow-fed rats, as well as significantly higher (*p* < 0.05) levels of RANK, DPD, TRAB 5b, and NTx. In a rat model, an exclusive whole or refined grain diet lowered bone turnover and mass.

## 1. Practical Application

Grains are a key caloric supply that may contribute to the development of chronic illnesses all over the world. The effect of grains in regulating the activity of osteoblasts and osteoclasts was investigated in this study.

## 2. Introduction

Bone is an active connective tissue that undergoes a continuous process of renovation [1], and in the process of maintaining plasma calcium homeostasis, bone remodeling is crucial. It is known that resorption leads to bone loss in bone disorders such as osteoporosis [2]. Age-related declines in bone mineral density (BMD) occur in both cancellous and cortical sites in both males and females; in females, this bone loss occurs after menopause, while in men it takes place at a later stage, after the age of 70 [3,4].

When facilitators such as a parathyroid hormone (PTH), interleukin 1 (IL-1), tumor necrosis factor (TNF), or prostaglandin (PGE-2) drive osteoblasts to release substances that cause osteoclasts to resorb bone, osteoblasts use their biological signaling role to mediate osteoclastic resorption [2]. Bone mass and bone mineral density (BMD) in cancellous and cortical sites of both genders gradually decrease with aging [4]. Changes in BMD, especially in the cancellous bone are sexually dimorphic (where rapid accelerated bone loss is only experienced by women, after menopause). Bone loss happens later in life in males than in females [4], with this condition increasing progressively with aging, especially in men (after the age of 70 years), particularly in cancellous bone [3].

RANK ligand (RANKL), osteoprotegerin (OPG), and NF-kB receptor activator (RANK) are the primary controllers of bone remodeling signaling. The most essential elements in the formation of osteoclasts from their progenitors are RANK and RANKL. RANK is a protein present on the surface of mature osteoclasts and their progenitors, that belongs to the TNF family. The primary function of RANKL is to keep osteoclasts alive by inhibiting apoptosis and promoting differentiation and activity. Because OPG prevents RANKL from binding to RANK, it has the opposite effect to RANKL [5].

This investigation prompted the discovery of a connection between contemporary, primarily western, food and several illnesses, particularly bone resorption. It is well-recognized that saturated fats and simple sugars account for a substantial percentage of daily calories in western nations. They are linked to metabolic disorders such as obesity, cardiovascular disease, and diabetes mellitus [6,7]. The adoption of flour and other grains into the human diet, which dates back ten thousand years, was also linked to an increase in infections, bone disorders including osteoporosis, kidney disease, metabolic disorders, higher neonatal mortality, and a shorter life span [7,8]. These osteoporotic changes can be explained by the malabsorption of nutrients, especially vitamin D and minerals such as magnesium and calcium, and also by an increase in the chronic inflammatory response that stimulates the production of bone-demineralizing cytokines, such as interleukins [9]. As a result of modern eating habits, chronic acidosis develops, leading to osteoporosis, bone fragility, and fractures. In people over the age of fifty, 53.2 percent of women and 20.7 percent of men can expect to have a fracture in their lifetime [10]. There also appears to be a link between osteoporosis and celiac disease, whether the latter has been diagnosed. According to a 2009 study, 13% of persons with osteoporosis tested positive for the gliadin antibody despite having no symptoms or indicators of celiac disease [11]. Undiagnosed celiac disease was discovered in 3.4 percent of participants with osteoporosis in Washington University Bone Clinic research, but just 0.2 percent of those without osteoporosis [12].

It is expected that when rats ingest grains as their primary source of energy, this would indirectly activate osteoclasts and elevate the RANK/OPG ratio. The current study’s objective was to study the effect of a grain-based diet on bone turnover indicators.

## 3. Material and Methods

### 3.1. Animals

For all investigations, thirty male Wistar Kyoto (WKY) rats (age: 12–14 weeks and weight: 200–300 g) were used. All rats came from Sultan Qaboos University’s Small Animal House, where they were kept under strict circumstances (23 degrees Celsius, 12:12 light–dark). The study followed the Declaration of Helsinki, and the methodology was certified by Sultan Qaboos University’s Ethics Committee with the registration number SQU/AEC/2018–2019/02.

### 3.2. Experimental Design

After one week of acclimating to the laboratory environment, the rats were randomly allocated to one of three groups. Each group consisted of ten rats. The Control Group (CON) consisted of rats that were fed 50 g of chow meal daily for twelve weeks. The Whole Grain Bread Consumption group consisted of rats who were given 50 g of whole grain bread daily for twelve weeks. The Refined Grain Bread Consumption group was fed 50 g of refined grain each day for twelve weeks [6]. The rats of this experiment were subjected to behavioral tests and the data of these tests were previously published in 2021.

Table 1 and Table 2 illustrate the macronutrients of the meal for the various groups.

### 3.3. Blood Collection and Biochemical Analysis

The rats were anaesthetized with sodium pentobarbital (i.p, 60 mg/kg) (Apollo Pharmaceuticals Api Manufacturers India Pvt. Ltd., Mumbai, India) by the end of week twelve. Blood samples (1 mL) were collected directly from the hearts and centrifuged at 3000 rpm for 10 min at room temperature. The serum was then collected and stored at −20 °C until the time of analysis. The following elements were then investigated using rat-specific ELISA kits: serum levels of testosterone, osteoprotegerin (OPG), Tartrate-resistant acid Phosphatase 5b (a bone resorption marker), cross-linked N-Telopeptide of type 1 collagen (NTXI) (a bone resorption marker), osteocalcin (OC) (a bone formation marker), alkaline phosphatase (ALP) (a bone formation marker), deoxypyridinoline (DPD) (a bone resorption marker), and Receptor Activator of Nuclear Factor Kappa B (RANK). The Elisa kits used were as follows: Cat. No. MBS2700368, Cat. No. MBS27101236, Cat. No. MBS2702692, Cat. No. MBS2700254, Cat. No. MBS2701838, Cat. No. MBS2506789, and Cat. No. MBS2704130, respectively (MyBioSource, San Diego, CA, USA).

### 3.4. Measurement of BMD (Bone Mineral Density) and BMC (Bone Mineral Content) of Right Tibia

The right hind limb of each rat was dissected at the hip joint after blood sampling, and the BMD and BMC of the right tibia were assessed using a LUNAR PIXI #50778 DEXA scan. The Lunar PIXImus is a fully integrated densitometer that estimates BMD and BMC using dual-energy X-ray absorptiometry (DEXA). It took four to five minutes to run the full scan on each rat sample.

### 3.5. Estimation of Tibial Bone Oxidative and Anti-Oxidative Parameters

The surrounding soft tissue was then cleaned from the tibia. The bone specimens were collected, and after weighing samples were crushed and homogenized in cool distilled water, then the samples were centrifuged at 3000 r.p.m. The supernatant produced was stored at –80 °C for later analysis. A colorimetric kit (Bio-Diagnostics, Dokki, Giza, Egypt) was used to analyze malondialdehyde (MDA) (CAT. No. MD 25 29), reduced glutathione (GSH) (CAT. No. GR 25 11), catalase (CAT) (CAT. No. CA 25 17), and superoxide dismutase (SOD) (CAT. No. SD 25 21) in the supernatant.

### 3.6. Statistical Analysis

The observed data were statistically analyzed using the GraphPad Prism statistical software tool (Version 8/Sydney, Australia). One-way ANOVA was performed, followed by Tukey’s post hoc test. Pearson correlation statistical analysis was performed for the detection of a probable significance between two different parameters. The information is presented in the form of a mean and standard deviation. Two-way ANOVA was performed to analyze the body weight during the 12 weeks of the experiments. When *p* < 0.05 is applied, the values will be significantly different.

## 4. Results

### 4.1. Grain Consumption Decreased OPG and Increased RANK

**Figure 1:**
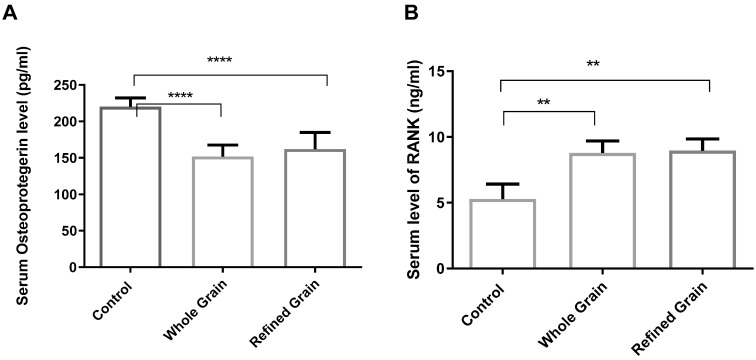
(**A**) Effect of whole grains and refined grains on serum levels of OPG. (**B**) Effect of whole grains and refined grains on serum levels of RANK. Data are expressed as mean ± SD of 10 rats. OPG: osteoprotegerin; RANK: Receptor Activator of Nuclear Factor Kappa B. ** *p* < 0.01, **** *p* < 0.00001.

As shown in Figure 1A,B, the WKY rats who were fed on refined and whole-grain bread both had a lower plasma level of OPG (*p* < 0.01) and a significantly higher level of RANK (*p* < 0.01) than the control rats. However, when comparing the rats fed with refined grain with those fed with whole grain, the difference in the levels of OPG and RANK was non-significant (*p* > 0.05).

### 4.2. Grain Consumption Increased Bone Resorption Markers

**Figure 2:**
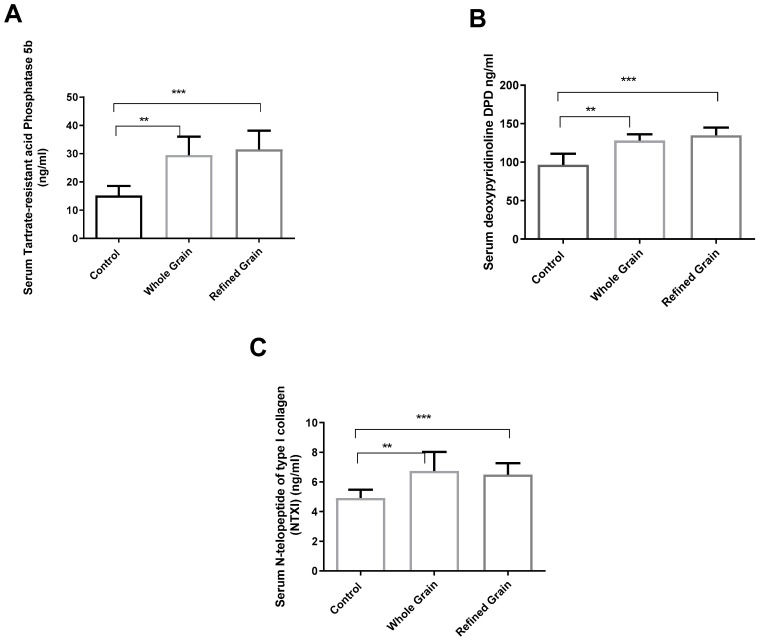
(**A**) Effect of whole grains and refined grains on serum levels of TRAP-5. (**B**) Effect of whole grains and refined grains on serum levels of DPD. (**C**) Effect of whole grains and refined grains on serum levels of NTXI. Data are expressed as mean ± SD of 10 rats. DPD: deoxypyridinoline; NTXI: N-telopeptide of type I collagen; TRAP-5b: Tartrate-resistant acid Phosphatase 5b. ** *p* < 0.01, *** *p* < 0.0001.

As shown in Figure 2A–C, the bone resorption markers (NTXI, TRAP 5b, and DPD) in both groups consuming grain were significantly higher (*p* < 0.01) than the markers in the control group rats. The differences between the two grain-fed groups, however, were less significant. The whole-grain consumption group had a higher serum level of N-telopeptide of type I collagen than those in the refined-grain group, but the difference was non-significant (*p* > 0.05). The refined grain group had higher deoxypyridinoline and Tartrate-resistant acid Phosphatase 5b levels than the whole grain group, but again the difference was non-significant (*p* > 0.05).

### 4.3. Grain Consumption Reduced OC and ALP

**Figure 3:**
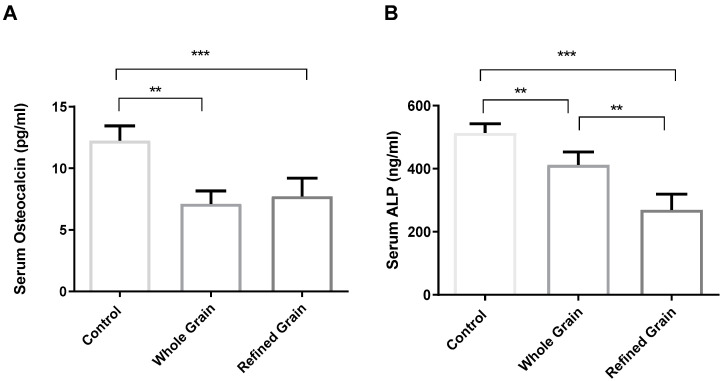
(**A**) Effect of whole grains and refined grains on serum levels of OC. (**B**) Effect of whole grains and refined grains on serum levels of ALP. Data are expressed as mean ± SD of 10 rats. OC: osteocalcin, ALP: Alkaline Phosphatase. ** *p* < 0.01, *** *p* < 0.0001.

As shown in Figure 3A,B, the consumption of both whole and refined grain resulted in the plasma levels of the bone formation biomarkers (OC and ALP) being significantly lower (*p* < 0.01) than the level of these two biomarkers in the control group rats. Interestingly, there was also a significant difference between the two grain-fed groups, with a significantly (*p* < 0.05) lower serum level of ALP in the refined grain group than in the whole grain group.

### 4.4. Effect of Grain on Bone Mineral Density (BMD) and Bone Mineral Content (BMC)

**Figure 4:**
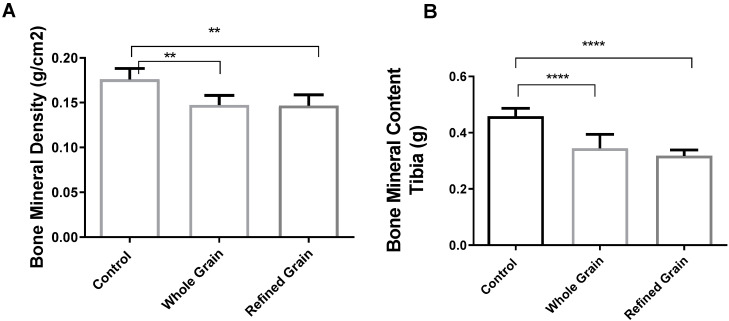
(**A**) Effect of whole grains and refined grains on bone mineral density (BMD). (**B**) Effect of whole grains and refined grains on bone mineral content (BMC). Data are expressed as mean ± SD of 10 rats. ** *p* < 0.01, **** *p* < 0.00001.

As shown in Figure 4A,B, the BMD and BMC were significantly lower (*p* < 0.01) in the grain consumption groups than in the control group.

## 5. Effect of Grains on Body Weight

**Figure 5:**
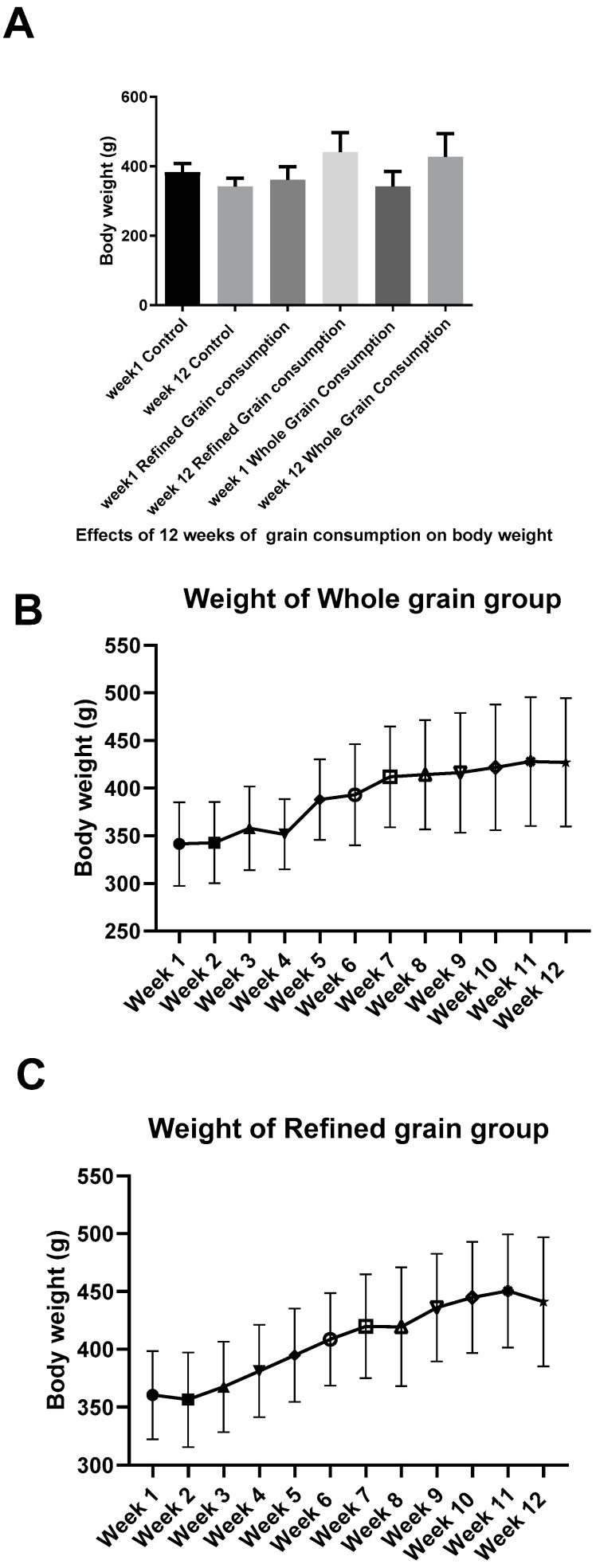
(**A**) Changes in body weight in the different groups. (**B**) Whole grain effects on body weight over 12 weeks. (**C**) Refined grain effects on body weight over 12 weeks.

Figure 5 showed that whole and refined grains consumption for 12 weeks increased the body weight significantly (*p* < 0.05) as compared to the control group. In addition, the control rats showed non-significant (*p* > 0.05) decrease in body weight.

### 5.1. Grains Increased MDA and Decreased GSH, SOD, and CAT

As shown in Table 3, feeding rats on the whole and refined grains resulted in significant changes to the oxidative stress parameters. There was a significantly higher MDA (*p* < 0.05) in the whole and refined grain groups than in the control group, and a significantly lower SOD, GSH, and CAT (*p* < 0.05).

### 5.2. Correlations between Different Parameters Studied

The serum OPG level and MDA (r = −0.958; *p* < 0.001) were negatively correlated, but a positive correlation was observed between serum levels of RANK and MDA (r = 0.8754; *p* < 0.001). 

## 6. Discussion

To our knowledge, this is the first study investigating the effect of grain consumption on bone turnover markers, BMD, and BMC. The findings of the study were clear as shown in Table 4. Firstly, a diet consisting entirely of either whole or refined grain decreased BMD and BMC in rats; secondly, the use of grain as a primary source of energy decreased OPG, OC, and ALP and increased RANK, NTXI, TRAP 5b, and DPD.

People in many parts of the world use grains, wheat, rice, and maize as their staple food. Current wheat flour is made from Triticum aestivum, composed of the weight of 70% carbohydrate, and 10% to 15% each of complex fiber and protein, with a lipid (generally phospholipids and polyunsaturated fatty acids) as a minor component. The starch in wheat flour is a complex carbohydrate consisting of 75% amylopectin, a chain of branching glucose units, and 25 percent amylose, a linear chain of glucose units. When grains are consumed, both amylopectin and amylose can be partially digested by salivary and pancreatic amylase. Amylase enzymes break down amylopectin into glucose monomers, but amylose is not completely digested and part of it ends up in the colon undigested. The complex carbohydrate amylopectin is quickly converted to glucose and absorbed into the circulation, causing a spike in blood glucose due to its rapid digestion [6,14]. 

The consumption of grains also contributes to both hyperglycemia and osteoporosis. Insulin resistance is linked to lipid peroxidation and a reduction in antioxidant enzyme activity [15]. The present study showed that rats that were fed with grains had a significant decrease in their GSH, CAT, and SOD, and an increase in their MDA (see Table 3). Other studies have also demonstrated a link between oxidative stress and osteoporosis, with increasing production of ROS and/or a decrease in the capacity of the antioxidants that are involved in the development of bone resorption. ROS stimulates the programmed cell death of osteocytes and osteoblasts, which suppresses mineralization and osteogenesis, and promotes the formation of osteoclasts. Excessive osteocyte apoptosis is also associated with oxidative stress, causing an imbalance in favor of osteoclastogenesis, leading to the augmented turnover of bone remodeling and bone resorption [16]. The current study indicates that the consumption of grains changed both bone antioxidant enzymes and ROS. Oxidative stress exacerbates a bone remodeling state that leads to an imbalance between the osteoclast and the osteoblast. This imbalance can lead to metabolic bone diseases and osteoporosis, which are characterized by low bone mineral density and a decrease in bone mass, making the bones weak and more prone to fracture [16,17,18].

Several other studies have corroborated our research findings. Baek [17] and his research group concluded that ROS stimulates the expression of M-CSF and RANKL and increases the RANKL/OPG ratio, leading to bone resorption and low bone mass in otherwise healthy women. Osteoblast differentiation, osteoclast activity, and mineralization are all affected by oxidative stress, whether directly or indirectly. Interestingly, another study in 2006 found that in older women the plasma level of antioxidants that are associated with osteoporosis significantly decreased [19], while a 2017 study on the role of antioxidants and OS demonstrated that the addition of H_2_O_2_ to cultures of human marrow mononuclear cells led to a higher number and activity of osteoclasts, as well as in the level of tartrate-resistant acid phosphatase [16].

Lifestyle, food intake, and daily activities, then, affect the mechanisms of bone formation and resorption [20]. The modern diet causes the blood’s pH to become less alkaline, which results in a predisposition to bone demineralization. The difficulty originates when acids present in ingested food need to be offset by calcium, and this stimulates the release of calcium from its stores. Though bones have a lot of stored calcium, the supply is not infinite, and this release of calcium will eventually result in osteopenia and osteoporosis, bone weakness, and fractures [21]. There have been several suggestions about how to counter this, and the consumption of calcium supplements is widespread. Unfortunately, however, this has not proved efficient at reversing and stopping bone resorption. A more efficient dietary method was put forward by Thorpe and his team who concluded that higher-frequency consumption of protein-rich foods was associated with reduced wrist fractures. This result supports the idea that adequate protein is important for bone health [22] and corroborates our findings that a protein-richer chow diet, presented in Table 1 and Table 2, was better for bone health than a grain diet, which is lower in proteins and may share in the pathogenesis of lower bone mineral density and content. One reason for this is that grain consumption causes acidification of the blood, with the amount of sulfuric acid released from wheat being greater than the same amount, per gram, released from meat [23]. In addition, the phosphorus present in cereal grains is stored in the form of phytic acid, which chelates minerals and decreases their bioavailability, with studies demonstrating that phytic acid particularly decreases the absorption of iron, zinc calcium, magnesium, and manganese [24]. Moreover, mg depletion may have a direct impact on bones (by lowering bone rigidity, increasing osteoclasts, and decreasing osteoblasts), as well as a secondary (indirect) effect (by interacting with PTH and vitamin D, boosting inflammation/oxidative stress and resulting in a bone loss effect) [25].

The bone is not a static structure; it is composed of dynamic tissue that is constantly forming and breaking away. The actions of osteoclastic and osteoblastic cells regulate the process of continual turnover (bone remodeling). One of the systems essential to bone metabolism and osteoclastogenesis is the RANKL/RANK/OPG pathway. The data in our study showed that feeding rats with whole and refined grains produced a significantly higher circulating level of RANK than that found in the control group rats. The grain diet was also associated with a significantly lower OPG level than the chow diet, with the differences in the grain-fed rats being related to chronic inflammation. Bone loss and osteoporosis in rheumatoid arthritis have been linked to the TNF-family member RANKL and its receptors (RANK) [26]. RANKL and RANK are considered the primary regulators for the development of osteoclasts. Intriguingly, Ginaldi and his colleagues [27] demonstrated that the binding of RANKL to its ligand receptors expressed on hematopoietic progenitors was followed by osteoclast differentiation and bone resorption. When RANK is turned on, it transmits signals to cells via tumor necrosis factor receptor-associated factors (TRAFs), specifically TRAF6. These RANK-associated molecules govern bone resorption, activation, survival, and differentiation of osteoclasts and dendritic cells via downstream pathways such as NF-kB, JNK/SAPK, p38, and Akt/PKB [27]. 

Systemic inflammation has been related to various physical and mental illnesses, such as metabolic syndrome, cardiovascular disease, cancer, autoimmune diseases, osteoporosis, schizophrenia, and depression [28]. It is known that systemic inflammation is associated with elevated plasma levels of proinflammatory cytokines such as interferons (IFNs), interleukin (Il)-1, Il-6, tumor necrosis factor-α (TNF-α), and C-reactive protein (CRP). CRP synthesis in the liver is induced by IL-1, IL-6, and TNF-, and is considered a sensitive indicator of systemic inflammation [29]. Several immunological and inflammatory illnesses in healthy people have shown a link between bone mineral density and circulating high sensitivity (hs) CRP levels, implying a connection between osteoporosis and subclinical systemic inflammation [30].

There is also a clear link between wheat consumption and chronic inflammation. Wheat has a protein composition that ranges from 7% to 22%, with gluten accounting for roughly 80% of the total protein in the grain [31]. Glutenins are polymers of individual proteins that are soluble in dilute acids and found in wheat proteins. Alcohol-soluble proteins present in cereal grains are known as prolamins, also known as gliadins in wheat. Gliadins are monomeric proteins that are classified as α/β-gliadins, γ-gliadins, and ω-gliadins [32]. The gliadin epitopes present in wheat gluten and its prolamins, which are produced from other grains including rye and barley, can cause chronic inflammation in the small intestine, leading to malabsorption and shrinkage of the intestinal villi. The primary processes that cause intestinal permeability and leaky gut include T-cell activation and elevated proinflammatory cytokines [6].

Several studies have investigated the effects of a gluten-free diet on bone mass density, or BMD. Interestingly, in a study conducted by Kotze and his research group bone mass density was slightly improved by a gluten-free diet, with low-impact fractures occurring in only one third of patients [33]. Similar findings were described by Pantaleoni et al.: after a 1-year gluten-free diet, participants had an increased BMD in the lumbar spine and femoral neck. In addition, links have been found between celiac disease and bone formation and resorption. A study by Silva et al. showed that 69% of Brazilian patients with celiac disease had low bone mineral density at diagnosis, the problem being more common in women over fifty [34]. In our research, the fact that OPG was significantly lower in the two grain-fed groups of rats than in the chow-fed control group could be attributed to gluten sensitivity. A relevant study from the University of Toronto examined the effect of increasing gluten ingestion from bread on the level of urinary calcium and found that the rate of calcium excretion in urine increased significantly, by 63%, in response to excess gluten intake. This elevated level of calcium excretion was also associated with an increase in bone resorption markers and induction of bone demineralization.

There are two classes of indicators of bone remodeling released from osteocytes and osteoclasts, as well as from the bone matrix. The first is the cluster of bone formation markers which includes procollagen type I N-terminal propeptide (PINP), alkaline phosphatase (ALP), bone-specific alkaline phosphatase, or osteocalcin. The second is the cluster of resorption markers which include C- or N- terminal telopeptide of type I collagen (CTX or NTX) and the pyridinolines that promote bone loss [35]. Our research found that grain-fed rats had significantly lower bone formation markers (OC and ALP) than chow-fed control-group rats, and significantly higher bone resorption markers (NTXI, TRAP 5b, and DPD). In clinical practice, an increase in the levels of tartrate-resistant acid Phosphatase 5b (TRAP 5b), deoxypyridinoline (DPD), and cross-linked N-telopeptide of type 1 collagen (NTXI) are considered strong markers for bone loss and decreased BMD (osteoporosis). TRAP 5b is a protein that is normally secreted by osteoclasts during bone resorption and is released in urine, thus reflecting osteoclast activity and numbers [36]. DPD, however, is a collagen-stabilizing molecule that crosslinks individual collagen peptides, and, similar to TRAP 5b, is also released in the urine [37]. NTXI is secreted when collagen degrades bone resorption. During osteoporosis, the levels of all these markers are significantly increased in the serum and urine of patients [38,39].

### Limitations

This research has three significant flaws. First, we could not explore whether gluten-free grain or bread intake would reduce or eliminate the harmful effects of the grain diet employed in our study. The second, is that we lacked the necessary equipment to assess gluten sensitivity. Thirdly, we could not measure RANKL in this experiment. Our future research goals aim to address these issues.

## 7. Conclusions

Reduced bone mass can indicate an imbalance between a structural requirement for calcium and phosphate and their biological requirement, which can signal metabolically active states such as inflammation. Inflammation and oxidative stress are driven by immunosenescence, osteoporosis, and other age-related illnesses. Lifestyle, including diet, affects both the systemic and the local inflammatory state. Grain consumption was found to increase RANK and decrease OPG, which can be associated with decreased BMC and BMD. Both whole and refined grains lead to a rise in oxidative stress, a decrease in calcium absorption, and a reduction in blood acidification, increasing bone resorption markers.

## Figures and Tables

**Table 1:** The macronutrients present in the chow, the whole grain, and the refined grain diets.

	Chow Diet (Con Group)	Whole Grain	Refined Grain
Carbohydrate %	63	68	71
Fat %	13	12	9
Protein %	24	20	20

**Table 2:** The macronutrients and micronutrients are present in whole grain and refined grain [13].

Nutrients	Whole Grain	Refined Grain
Calories from Protein %	20	20
Calories from Total lipids %	12	9
Calories from Carbohydrates %	68	71
Dietary fiber (g/kg)	115	19
Starch and sugar(g/kg)	700	830
Zn (µg/g)	29	8
Fe (µg/g)	35	13
Magnesium (mg/g)	1.38	0.22
Vitamin B_6_ (mg/g)	7.5	1.4
Folate (mg/g)	0.57	0.11
Ferulic acid (mg/g)	0.5	0.04
Vitamin A (µg/g)	32.8	5.7

**Table 3:** Effect of whole grains and refined grains on MDA, GSH, SOD, and CAT in the tibia of rats.

	Control	Whole Grain	Refined Grain
MDA (nmol/g tissue)	8.21 ± 0.14	11.34 ± 0.16 *	12.61 ± 0.18 * #
GSH (mg/g tissue)	6.42 ± 0.12	4.15 ± 0.11 *	4.75 ± 0.23 *
SOD (U/g tissue)	54.14 ± 3.14	27.14 ± 4.51 *	32.14 ± 5.61 *
CAT (U/g tissue)	0.58 ± 0.02	0.27 ± 0.08 *	0.39 ± 0.05 *

Effect of whole grains, refined grain on MDA, malondialdehyde; GSH, glutathione; SOD, superoxide dismutase; and CAT, catalase. Data are expressed as mean ± SD of 10 rats. * *p* < 0.05 versus control, # *p* < 0.05 versus whole grains.

**Table 4:** Effect of whole grains and refined grains on serum levels of RANK, OPG, TRAP-5, DPD, NTXI, osteocalcin, and ALP.

	Control	Whole Grain	Refined Grain
RANK (ng/mL)	5.287 ± 1.134	8.78 ± 0.90 *	8.95 ± 0.88 *
OPG (pg/mL)	220.1 ± 12.18	151.7 ± 15.86 *	161.9 ± 22.94 *
TRAP 5b (ng/mL)	15.19 ± 3.381	29.46 ± 6.575 *	31.53 ± 6.625 *
DPD (ng/mL)	96.66 ± 14.33	128.1 ± 8.104 *	134.9 ± 9.980 *
NTXI (ng/mL)	4.923 ± 0.5537	6.739 ± 1.292 *	6.496 ± 0.7727 *
Osteocalcin (pg/mL)	12.24 ± 1.199	7.106 ± 1.054 *	7.719 ± 1.47 *
ALP (ng/mL)	513.1 ± 29.48	412.3 ± 40.70 *	269.6 ± 49.37 * #

OPG: osteoprotegerin, RANK: Receptor Activator of Nuclear Factor Kappa B, DPD: deoxypyridinoline, NTXI: N-telopeptide of type I collagen, TRAP-5b: Tartrate-resistant acid Phosphatase 5b, and ALP: Alkaline Phosphatase. Data are expressed as mean ± SD of 10 rats. * *p* < 0.05 versus control, # *p* < 0.05 versus whole grains.

## Data Availability

Data is available with corresponding author.
